# Impact of treating dental caries on schoolchildren’s anthropometric, dental, satisfaction and appetite outcomes: a randomized controlled trial

**DOI:** 10.1186/1471-2458-12-706

**Published:** 2012-08-29

**Authors:** Heba A Alkarimi, Richard G Watt, Hynek Pikhart, Amal H Jawadi, Aubrey Sheiham, Georgios Tsakos

**Affiliations:** 1 King Fahad Armed Forces Hospital (KFAFH), P.O. Box 54146, Jeddah, 21514, Saudi Arabia; 2 Department of Epidemiology and Public Health, University College London, London, UK

**Keywords:** Dental caries, Child, Anthropometry, HAZ, WAZ, Appetite, Pain, Sepsis, Satisfaction

## Abstract

**Background:**

There are no randomized controlled trials to assess the impact of treating dental caries on various aspects of children’s health. This study was conducted to assess the impact of dental treatment of severe dental caries on children’s weight, height and subjective health related outcomes, namely dental pain, satisfaction with teeth and smile, dental sepsis and child’s appetite.

**Methods:**

The study was a community-based, randomized, controlled trial in schoolchildren aged 6-7 years with untreated dental caries. Participants were randomly assigned to early (test) or regular (control) dental treatment. The primary outcome was Weight-for-age Z-score. Secondary outcomes were Height-for-age and BMI-for-age Z-scores, dental pain, dental sepsis, satisfaction with teeth and child’s appetite.

**Results:**

86 children were randomly assigned to test (42 children) and control (44) groups. Mean duration of follow-up was 34.8 (±1.1) weeks. There were insignificant improvements in anthropometric outcomes between the groups after treatment of caries. However, treated children had significantly less pain experience (P = 0.006) (OR 0.09, [0.01-0.51]) and higher satisfaction with teeth (P = 0.001) (OR 9.91, [2.68-36.51]) compared to controls. Controls had significantly poorer appetites (P = 0.01) (OR 2.9, [1.24-6.82]) compared to treated children. All treated children were free of clinical dental sepsis whereas 20% (9 of 44) of controls who were free of sepsis at baseline had sepsis at follow-up.

**Conclusions:**

Although dental treatment did not significantly improve the anthropometric outcomes, it significantly improved the dental outcomes and children’s satisfaction with teeth, smile and appetite. This is the first study to provide evidence that treatment of severe dental caries can improve children’s appetite.

**Trial registration:**

Effect of Dental Treatment on Children's Growth. Clinical Trial Gov ID# NCT01243866

## Background

Dental caries is one of the most prevalent chronic diseases of children worldwide. In many countries, most dental caries in young children is untreated [1]. Dental caries is generally not life-threatening. However, the burden of untreated caries is very high when the context of general and emotional health and treatment costs is considered [2-4]. Untreated severe caries can lead to pulpitis and sepsis, extending to the supporting tissues and sometimes cause serious complications such as cellulitis and brain abscesses [5]. Caries experience is associated with poor child growth and low weight gain [6-9], increased treatment time and cost [10,11], higher risk of hospitalization [11,12], days missed from school and work and compromised school performance [13].

Studies suggest that children’s growth improved by eliminating dental pain and sepsis that negatively affected children’s ability to eat and sleep [14,15]. Unfortunately, the studies have many methodological limitations. The only randomized controlled trial (RCT) on the aforementioned relationship used anthropometric outcomes as the only measures of the effect of treatment of caries on children’s health [16]. Yet, no RCT has addressed the impacts of treatment of severe caries on children using clinical, anthropometric and subjective health outcomes. Ethical considerations for doing clinical trials on children is one explanation for the lack of RCTs. Producing a comparable control group that will not receive dental treatment from hospital-based sampling is an ethical dilemma. Therefore, to overcome this problem, some studies used either non-randomly assigned caries-free controls [6] or growth references as a proxy [17].

Consequently, there is a clear need for innovative approaches to address the absence of robust evidence of the impact of treating severe dental caries on children’s health and well-being. We hypothesized that treatment of severe caries would significantly improve children’s clinical, anthropometric and subjective health outcomes. A randomized controlled trial was carried out with the objective of assessing the impact of dental treatment of severe dental caries on children’s weight, height and subjective health related outcomes, namely self-reported dental pain, self-reported satisfaction with teeth and smile, clinical dental sepsis and parental-report of child’s appetite 6 months post-dental intervention. To adequately address ethical concerns, a community-based sample was used. Eligible children were selected from their schools and not from hospitals and provided either with ‘early’ or ‘regular’ dental treatment. All children would normally be on a waiting list of at least 8 months for ‘regular’ dental treatment. So the treatment of the controls would not be delayed. The control children did receive the same dental treatment as the test group, but six months later. That minimizes the potential ethical considerations about delaying treatment for control group.

## Methods

### Participants

Eighty six schoolchildren aged 72 to 95 months with severe dental caries were selected from schoolchildren attending military primary schools and who were therefore eligible for dental treatment at King Fahad Armed Forces Hospital (KFAFH), Jeddah, Saudi Arabia. This population of schoolchildren was selected for the study as they had the highest prevalence of dental caries in Jeddah [18]. All Grade 1 schoolchildren were invited. 417 of 436 children were clinically examined (dental examination and anthropometric measurements) and had face-to-face interviews. 122 of the 417 examined schoolchildren were identified as potentially eligible for the trial and referred to KFAFH for further screening. Only children with severe dental caries were included. Severe dental caries was defined as having at least 2 teeth with pulpal involvement at enrolment. Pulpal involvement was used as a criterion because teeth with infected pulps negatively affect children’s eating and sleeping abilities [14,15,19] and are also linked to higher levels of inflammation, which has been shown to affect immunity [20-22], contribute to anaemia [23] and potentially lead to growth failure [24,25].

The following groups were excluded; those with illnesses that adversely affect growth; children needing urgent dental treatment; children on regular nutrition supplements and anemic children with hemoglobin levels lower than 11.0 g/dl.

### Study design

This community-based, randomized trial was undertaken between February 2007 and January 2008. Children were randomly assigned to early (test) or regular (controls) dental treatment. Those in test and control groups received dental treatment according to standard local practices but test children were treated approximately 6-month before controls so that a comparison could be made between treated and untreated children. Simple randomisation was used as eligibility criteria were highly restricted to keep the study subjects more homogenous. The randomisation using tables of random numbers was done between April and May 2007, after screening was completed and was independent of the investigator. Blinding of the treating paedodontist and participants was not feasible given the nature of the study. The design, conduct and reporting of this study complied with the CONSORT (Consolidated Standards of Reporting Trials) statement [26].

### Dental intervention

Test children were scheduled for comprehensive dental treatment over a 2-month period (from May to June 2007). Non-restorable grossly decayed teeth and teeth with signs of radiographic pathology and pathological mobility were extracted. Extensive carious lesions such as those with loss of more than two-thirds of the marginal ridge were treated with stainless steel crowns. Teeth with inflamed coronal pulp and healthy radicular pulp tissue were treated by ferric sulphate pulpotomy and crowned with stainless steel crowns. Carious teeth with small lesions with no pulpal involvement were treated with dental fillings.

All test children had their last dental treatment visit in the trial within the last 2 weeks of the second treatment month. At their last visit children were examined to ensure that they were free of dental caries and dental infections. The follow-up survey was scheduled for approximately 6-month after each child’s last dental visit to ensure all children were examined at exactly the same interval between end of treatment and follow-up. Control children did not receive any dental treatment in the period when the test children were treated, unless they had toothache. In that case they were treated for pain by extraction or filling but did not have comprehensive dental treatment. They did receive the same dental treatment as the test group, six months later than the test group.

### Outcomes

Outcome measures were assessed at baseline and 6 months post dental intervention. The primary outcome variable was Weight-for-age Z-score (WAZ). Secondary outcomes included Height-for-age Z-score (HAZ), BMI-for-age Z-score (BAZ) [27], dental pain, dental sepsis, satisfaction with teeth and smile and child’s appetite.

### Measurements

Data collection at baseline for children and their parents followed a standardized protocol. All anthropometric and dental examinations and the face-to-face interviews of the baseline and follow up survey were done at children’s schools, away from the dental surgery, by a trained team who were unaware of the study rationale. Nutritional status was assessed using WHO AnthroPlus software that holds the WHO Reference 2007 for 5-19 years old children [28]. Anthropometric measurements were performed according to the Food and Nutrition Anthropometric Indictors Measurement Guide [29]. Measurements for height and weight were taken to the nearest 0.1 cm and 0.1 kg, respectively. Height was measured with child standing without shoes using a portable Harpenden pocket stadiometer (Chasmors Ltd, London, UK). Weight was measured with the child standing wearing light clothes and not wearing shoes using a pre-calibrated digital Seca scale (Model 767, Hamburg, Germany).

Clinical dental sepsis was defined as “dental abscess presenting as localized swellings or draining sinuses adjacent to carious tooth” [30]. To assess subjective health outcomes, valid questionnaires for both parents and children were used and translated into Arabic. Clarity, suitability and cultural adaptability of the Arabic version of the questions were tested in the pilot study. Children were asked standard questions on pain and satisfaction with teeth and smile [14,15,19,31]. For subjective assessment of appetite, validated questions were answered by parents of children [32,33]. Reproducibility of data was checked by repeating measurements in 12% of the sample.

### Ethics

The trial was approved, managed and monitored by the Research and Ethics Committee in KFAFH. Monitoring was continued throughout the trial to ensure protocol adherence. Informed consent was obtained from all parents of children.

### Sample size

There have been no published randomized controlled trials on the effect of dental treatment on children’s anthropometric and subjective health outcomes at the time of the trial. The calculation of child’s anthropometric changes was based on weight gain as it needs less follow-up time than height gain [25]. On the basis of available information and expert opinion, it was assumed that the clinical significance was 0.25 Z-score and the common within-group standard deviation estimated to be 0.39. The criterion for significance (alpha) was set at 0.05. With the proposed sample size of 40 for both test and control groups, the study would have power of 80% to yield a statistically significant result. The sample was increased by 10% to account for any deviations in protocol, thus 88 children (44 per group) were enrolled.

### Statistical analysis

All analyses were carried on an intention-to-treat basis. The baseline observation carried forward (BOCF) analysis was used to replace missing data [34]. Characteristics of the two groups were compared using Chi-square or Fisher’s exact test for categorical variables and *t* tests or Mann–Whitney *U* test for continuous variables. Categorical outcomes with ordered responses such as child’s appetite, were analyzed using a Chi-square test for trend. Analysis of covariance (ANCOVA) models were used with the baseline assessment score as the covariate. Odds ratios (OR) with 95% Confidence Intervals (CI) were calculated to measure treatment effects for secondary outcomes. To estimate the effect of dental treatment the secondary categorical outcomes were grouped into ordered categories of 1. improved, 2. no change and 3. worsen. SPSS (version 15) was used for all analyses.

## Results

**Figure 1:**
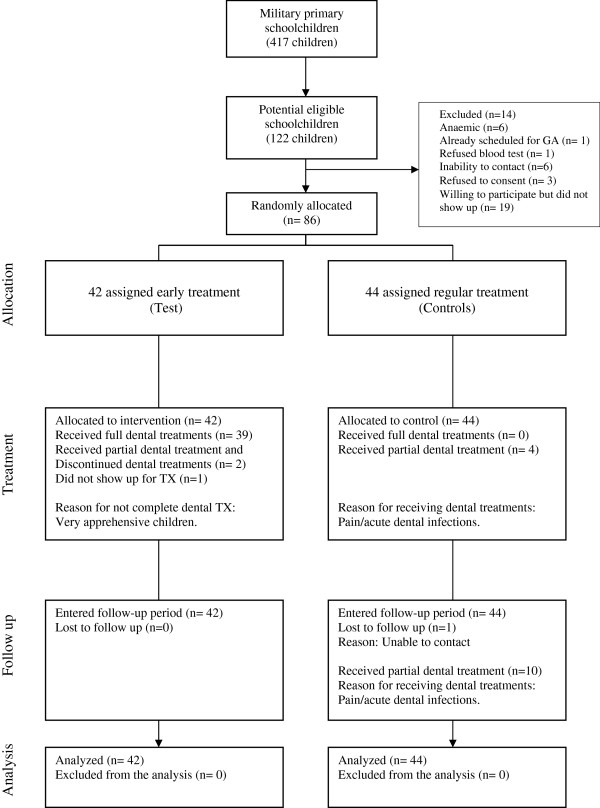
CONSORT flow chart of participants.

42 children were randomly allocated to test and 44 to control group (Figure 1). Of the 42 test children, 39 (92.8%) received the planned comprehensive dental treatment, and 3 did not. The reasons for not receiving the treatment were fear of dental treatment (2 cases) and busy parent (1 case). No child had adverse effects of dental treatment. 14 (31.8%) controls received some emergency dental treatment during the study period. There were no significant differences between the study groups in baseline characteristics (Table 1), with the exceptions of mother’s educational level (higher in controls) and child’s appetite (poorer in test children). These differences were taken into account in the ANCOVA analyses.

**Table 1:** Baseline characteristics of participants

**Characteristic**	**Early treatment (Test), n = 42**	**Regular treatment (Controls), n = 44**	**P**
**Age (months)**			
Mean (SD)	81.4(4.7)	82.0(4.6)	0.45
**Sex,** n (%)			
Male	16(38.1)	16(36.4)	0.83
Female	26(61.9)	28(63.6)	
**Father’s educational level,** n (%)			
Illiterate/Primary school	4(9.6)	3(6.8)	0.88
Secondary/High school	29(69.0)	32(72.7)	
University/postgraduate	9(21.4)	9(20.5)	
**Mother’s educational level,** n (%)			
Illiterate/Primary school	20(47.6)	11(25.0)	0.04
Secondary/High school	16(38.1)	23(52.3)	
University/postgraduate	6(14.3)	10(22.7)	
**Family income,** n (%)			
<7000 Reyals Saudi Reyal	10(23.8)	9(20.5)	0.61
7001-10000 Reyals	16(38.1)	16(36.3)	
>10000 Reyals	16(38.1)	19(43.2)	
**Mean height ‘cm’** (SD)	115.16(4.64)	115.29(4.67)	0.74
**Mean weight ‘kg’** (SD)	19.54(2.62)	20.00(2.17)	0.48
**Mean HAZ** (SD)	−0.89(0.84)	−0.92(0.83)	0.94
**Mean WAZ** (SD)	−0.93(0.98)	−0.78(0.80)	0.64
**Mean BAZ**(SD)	−0.58(1.07)	−0.32(0.76)	0.34
**Dental pain,** n (%)			
Yes	9(21.4)	14(31.8)	0.39
No	33(78.6)	30(68.2)	
**Dental sepsis,** n (%)			
Yes	9(21.4)	11(25.0)	0.89
No	33(78.6)	33(75.0)	
**Satisfaction,** n (%)			
Satisfied	24(57.1)	26(59.1)	0.85
Dissatisfied	18(42.9)	18(40.9)	
**Child’s appetite,** n (%)			
1 (High)	6(14.3)	11(25.0)	0.03
2	17(40.5)	23(52.3)	
3 (Low)	19(45.2)	10(22.7)	
**Mean Hb (g/dl)** (SD)	12.4(0.96)	12.5(0.79)	0.37
**Low birth weight,** n (%)			
Yes	12(28.6)	17(38.7)	0.82
No	30(71.4)	27(61.3)	
**Median of pulpal involvement**	6.0	6.0	0.91
**Median dmft**	9.5	9.0	0.86

There was a 98.8% (n = 85) overall response rate at the 6-month follow-up after treatment of the test children. Follow-up was completed for 42 test children (100%) and 43 controls (97.7%). The mean time interval between baseline and follow-up measures was 34.8 weeks (±1.1). There was no difference between test and control children in mean time difference between baseline and follow-up measurements (34.7 weeks versus 34.8 weeks P = 0.68). Mean time interval between baseline scores and completion of dental treatment for test group was 9.3 weeks (±2.7).

At baseline, test and control children were similar. At follow-up, test children had non-significant improvements in their mean WAZ, HAZ and BAZ compared to controls (p>0.05). In contrast, WAZ and BAZ of controls were worse than their scores at the baseline. All these differences were not significant (Table 2). Furthermore, adjustment for anthropometric pre-intervention scores did not considerably affect these findings. There was no significant difference in mean WAZ, HAZ and BAZ between the test and control children after adjusting for pre-intervention scores and all variables that showed significant differences between the groups at baseline (child’s appetite and mother’s educational level). However, test children had a larger improvement in all anthropometric outcomes compared to controls (Table 2).

**Table 2:** Mean change (95%CI) from baseline at 6 months of WAZ, HAZ and BAZ outcomes, by treatment groups

**Anthropometric outcomes**	**Early treatment (Test) n = 42**	**Regular treatment (Controls) n = 44**	**Mean difference**^**1**^	**Mean difference**^**2**^
	**Unadjusted mean change from baseline (95% CI)**	**Unadjusted mean change from baseline (95% CI)**	**(Test-Control) (95% CI; P)**	**(Test-Control) (95% CI; P)**
**WAZ**	0.09	−0.03	0.10	0.10
	(0.23, -0.07)	(0.04,-0.12)	(-0.07, 0.27; 0.23)	(-0.07, 0.27; 0.27)
**HAZ**	0.07	0.06	0.02	0.02
	(0.12,0.04)	(0.09,0.02)	(-0.03,0.07; 0.46)	(-0.04,0.06; 0.69)
**BAZ**	0.03	−0.12	0.12	0.13
	(0.03, -0.21)	(-0.01,-0.23)	(-0.13, 0.36; 0.37)	(-0.14,0.38; 0.35)

^1^ Adjusted for group types and pre-intervention score.

^2^ Adjusted for group types, pre-intervention score, mother’s educational level and child’s appetite.

At follow-up, no child in the test group reported worsening of self-reported dental pain or clinical sepsis while some children in the control group did report worsening. 14 out of 18 test children who were dissatisfied at baseline with their teeth and smile became satisfied with those features versus 5 out of 18 controls (P = 0.01). Test children had significantly higher odds of satisfaction with teeth and smile compared to controls. Test children reported poorer appetite compared to controls at baseline (P = 0.03). At follow-up, test children showed improvement in their appetite compared to controls (P = 0.01). In summary, test children showed significant reductions in dental pain and clinical dental sepsis, and improvement in satisfaction with teeth and smile and in their appetite, compared to controls (Table 3). These differences did not change after controlling for other covariates (child’s appetite and mother’s educational level) included in the final models (Table 4).

**Table 3:** Changes in secondary health outcomes between baseline and follow-up stages, by treatment groups

**Secondary health outcomes**	**Early treatment (Test)n = 42**	**Regular treatment (Controls)n = 44**	**P**
**Dental pain**			
Improved	7	11	<0.001
No change	35	22	
Worsen	0	11	
**Dental Sepsis**			
Improved	9	9	0.005
No change	33	26	
Worsen	0	9	
**Satisfaction**			
Improved	14	5	0.01
No change	27	32	
Worsen	1	7	
**Child’s appetite**			
Improved	20	10	0.01
No change	19	22	
Worsen	3	12	

**Table 4:** Adjusted regression models for secondary subjective health outcomes

	**Adjusted**^**1**^	**P**	**Adjusted**^**2**^	**P**
**Dental pain**				
OR (95% CI)	0.10(0.02,0.49)	0.005	0.09(0.01,0.51)	0.006
**Satisfaction**				
OR (95% CI)	8.89(2.55, 31.00)	0.001	9.91(2.68, 36.51)	0.001
**Child’s appetite**				
OR (95% CI)	2.98(1.28,6.95)	0.01	2.91(1.24,6.82)‡	0.01

^1^ Adjusted for group types and pre-intervention scores.

^2^ Adjusted for group types, pre-intervention scores, mother’s educational level and child’s appetite.

‡Adjusted for group types, pre-intervention scores and mother’s educational level only as appetite pre-intervention scores already taken into account in adjustment^1^.

## Discussion

Although dentally treated children showed improvements in WAZ, HAZ and BAZ while there were deteriorations in WAZ and BAZ and smaller improvements in HAZ in controls, the differences were not statistically significant. There was a statistically significant reduction in dental pain and dental sepsis and improvement in satisfaction with teeth and smile and child’s appetite in treated children compared to controls. This is the first RCT reporting changes in the abovementioned dental and quality of life outcomes from both children’s and parent’s points of view. Previous studies depended solely on parent’s responses [14,15,17,19].

A novel aspect of this study is the design that adequately addressed ethical and methodological challenges involved in this research. One ethical concern was how to find and recruit children for the RCT as some would not receive dental treatment immediately after recruitment. It is deemed unethical to recruit children from emergency dental rooms or children attending regular dental treatment and delaying dental treatment for them if they were assigned to the control group. In addition, children seeking dental treatment would have higher dental functional limitations compared to children attending schools and may need urgent dental treatment. In the current study, children were recruited from their schools and provided with either early or regular dental treatment. No attempts were made to delay dental treatment for the controls. Controls were given direct access to the study dentist if they had a dental emergency; indeed, 31% of them received emergency treatment during the study period.

Methodological concerns such as the validity of the weight measures were also considered. To improve the validity of anthropometric measures, all children were weighed at school at the same time of day (7:30 – 8:50 am) and in the same relation to their eating time (before morning break). Second, reproducibility of data was checked by repeating measurements in 12% of the sample. Third, the new WHO growth references were used as they are valid worldwide, irrespective of ethnicity and socio-economic status [27]. Fourth, time difference between baseline and follow-up measurements was minimal between groups to ensure the validity of the measurements and to increase the accuracy of the growth rates comparison.

The comparison of the present study with other studies on the relationship between caries and anthropometric outcomes presents certain difficulties. First, previous studies selected their samples from hospitals, not from a community-based population [6,17,18]. Recruiting young children from hospitals suggests they had high dental impacts that may be combined with high functional limitations. In this Saudi study, children were recruited from schools. Few children had functional limitations that prevented them from eating or sleeping. Second, a control group was only used in two studies that reported conflicting results [6,16]. The frequently quoted Acs’s study [6] used non-randomly assigned caries-free children as controls. The weight percentiles of test children were significantly lower than that of caries-free controls at baseline. This indicates that test and control groups were not comparable at baseline. In fact, 13% of children in the test group in the Acs study satisfied the failure to thrive criteria as they had very low weight measures. The findings of the present study are in agreement with van Gemert-Schriks’s randomized controlled study that reported insignificant changes in mean anthropometric outcomes of dentally treated children compared to untreated controls.

After treatment, children reported significantly lower levels of dental pain and dissatisfaction with teeth and smile than untreated controls; findings consistent with previous non-random studies. Treated children also had no sepsis compared to untreated controls. This is in agreement with findings from a British study [31], where the risk of dental sepsis increased with number of untreated carious teeth. In addition, treated children had a significantly increased likelihood of having improved appetite compared to untreated controls. As this study was a community-based RCT with a very high retention rate, assessors were blinded to group identity and selection criteria were restricted to ensure homogeneity and all measures were standardized and reliable, it appears that a real change occurred in the secondary health outcomes due to the dental intervention. Study results are generalizable to healthy children with severe dental caries and without severe functional limitations. Generalizability of this study is enhanced by the community-based sampling.

The study has some limitations. First, the follow-up period was only 6 months and incorporated a single follow-up point, which may not be entirely representative of the pattern of growth changes in children aged six and seven. More importantly, this short-term follow-up period may partly explain the lack of differences between test and control groups in terms of anthropometric outcomes. Significant differences between the groups may have been observed if a longer follow-up period was possible as the direction of change in anthropometric outcomes was in line with the hypothesis. This suggestion is supported by the observed significant differences between treated and untreated groups in other outcomes such as elimination of pain and sepsis and increased appetite that could be viewed as the more immediate effects of the intervention. It seems reasonable to assume that these improvements might precede improvement in children’s growth. However, delaying dental treatment of controls beyond six months was regarded as unethical, because control children had severe dental caries and their treatment could not be delayed to accommodate the ideal requirements for our study. Second, the power of the study can be questioned. The calculation of the sample size may not be appropriate as the estimation of the required number was calculated using expert opinion and an uncontrolled study in an industrialized country. Nevertheless, the fact that a difference was detected in all subjective secondary outcomes suggests that the RCT is robust enough to detect differences. Third, complete blinding of the trial was not feasible. Blinding of data collectors was implemented to avoid bias due to different assessments of the outcomes.

## Conclusions

Our findings show that treating severe dental caries in this child population significantly reduced dental pain, dental sepsis, dissatisfaction with teeth and smile and poor appetite. Differences between intervention and control groups in terms of children’s growth were not statistically significant, although they were in the expected direction. A further follow-up is planned and should provide additional information on the long-term effects of dental treatment on children’s anthropometric outcomes.

## Competing interests

The authors declare that they have no competing interests.

## Authors’ contributions

The authors’ responsibilities were as follows—HAA, AS, GT, HP, RGW conceived the study concept and design. HAA, HP, GT contributed to data analysis and interpretation. AHJ participated in the treatment and patient management. HAA wrote the first draft of the paper. AS, RGW, GT, AHJ, HP contributed to the critical revision of the manuscript for important intellectual content. All authors read and approved the final manuscript.

## Pre-publication history

The pre-publication history for this paper can be accessed here:

http://www.biomedcentral.com/1471-2458/12/706/prepub
